# THE UNIVERSITY OF NEBRASKA OMAHA DEPARTMENT OF GERONTOLOGY: LOOKING FORWARD TO THE NEXT 50 YEARS

**DOI:** 10.1093/geroni/igae098.0840

**Published:** 2024-12-31

**Authors:** Christopher Kelly

**Affiliations:** University of Nebraska Omaha, Omaha, Nebraska, United States

## Abstract

In 2023, the University of Nebraska Omaha (UNO) Department of Gerontology celebrated its 50 year anniversary. Since 1973, the department has been the primary provider of gerontological education in the state, offering Bachelor’s, Master’s and Doctoral degrees in gerontology. In this presentation, we will discuss how UNO Gerontology has grown its programs, particularly through partnerships with public and private stakeholders at the national, state, and local levels, in order to train the professionals who work with and on behalf of older Nebraskans. We will also describe how we expect these partnerships to evolve to meet the needs of our growing aging population.

